# Fabrication of flexible self-powered humidity sensor based on super-hydrophilic titanium oxide nanotube arrays

**DOI:** 10.1038/s41598-020-70031-z

**Published:** 2020-08-03

**Authors:** Elham Farahani, Raheleh Mohammadpour

**Affiliations:** 1 Department of Physics, Sharif University of Technology, 11155-9161 Tehran, Iran; 2 Institute for Nanoscience and Nanotechnology, Sharif University of Technology, 14588-89694 Tehran, Iran

**Keywords:** Nanoscience and technology, Nanoscale materials, Nanowires

## Abstract

Stable and flexible super-hydrophilic nanotubular-based titanium oxide electrode has been utilized as the active electrode of self-powered humidity sensor. TiO_2_ nanotubular electrodes fabricated through anodization method and utilized in combination with Kapton electrode as the triboelectric nanogenerator (TENG). Vertical contact-separation mode TENG performance has been examined in various range of frequencies and the maximum output voltage and current more than 300 V and 40 μA respectively with maximum power of 1.25 ± 0.67 mW has been achieved at 4 Hz. The fabricated TENG has been employed as the active self-powered humidity sensor. Super-hydrophilic feature of TiO_2_ nanotubes resulted in full absorption of water molecules, and noticeable decrease in charge transfer across two triboelectric materials upon increasing humidity. The TiO_2_-based TENG sensor was exposed to various relative humidity (RH) and the results showed that by increasing the humidity the output voltage and output current decreased from 162.24 ± 35.99 V and 20.4 ± 4.93 μA at RH = 20% to 37.92 ± 1.54 V at RH = 79% and 40.87 88 6.88 ± 1.7 μA at RH = 84%, respectively, Which shows the responsivity more than 300%. This method of measuring humidity has a simple and cost-effective fabrication that has various applications in many fields such as industry and medicine.

## Introduction

Limitation of energy resources around the world and environmental pollutions have attracted researchers' attention toward harvesting energy^1^. Among various energy sources (such as chemical, geothermal or solar) mechanical energy is not only environmentally friendly but also available at any time and place and is the most abundant energy in the environment around us^1–3^. Converters of mechanical energy to electricity have different types and include piezoelectric, electromagnetic and triboelectric nanogenerators^4–6^. Triboelectric nanogenerator (TENG) discovered in 2012 that works by transferring the surface charge between two dissimilar materials whereas they are in physical contact with each other. After the separation of the two surfaces via environmental mechanical energy, an electrical potential difference is induced and producing an electrical current^4,6–8^. TENG is affordable and lightweight and can be fabricated through easy fabrication process with variety of structures and highly efficient performance^4,7–11^.

Humidity detection and measurement are required for possessing better life. In recent years, they are employed in many fields such as meteorology, medical and agricultural equipment, biotechnology, air conditioning, commercial and defence airplane, fabric industries, quality monitoring food and the environment^12–16^. All of these sensors require electrical power to operate. Batteries due to problems such as limited life span, heavy weight and environmental damage cannot provide permanent energy demand, so considerable research has been done in the field of fabricating self-powered sensors. Self-powered sensors are suitable options because of their comfortable monitoring mechanism, easy construction and affordable through energy storage^2,17^. Humidity sensors can be classified into three main categories: polymeric, composite and ceramic materials. Ceramic moisture sensors have relatively high chemical, mechanical and thermal stability and they contain porous metal oxides such as SnO_2_, ZnO, WO_3_, Al_2_O_3_, TiO_2_, SiO_2_, In_2_O_3_^18–20^. Among this ceramics, TiO_2_ is very beneficial for humidity sensing due to Ti^3+^ defect sites for adsorption of water molecules, significant hydrophilicity and surface roughness. In addition, high surface-to-volume ratio and the nano-porous structure, which is due to its nanoscale grain boundaries, can provide a large surface area and create high sensitivity to water vapor^21–23^. Anatase phase TiO_2_ can more easily desorb physisorbed water molecules rather than the rutile phase^18,24^. Among the different types of nanostructured titania, TiO_2_ nanotube arrays is a good material for usage in humidity sensors due to highly uniform structure and controllable pore sizes. The relatively high surface-to-volume ratio of TiO_2_ nanotubes, due to the large surface of the interior and exterior, results in the production of higher response sensors in comparison to TiO_2_ thin films having only the outer surface^25–27^. Anodization is one of the simplest and most effective methods of fabrication. The response and recovery time depend on the thin nanotube walls that is easily adjusted in the anodization method by controlling the electrolyte composition and the applied voltage^25,26^. Liang's group fabricated TNT-based FETs by anodization that showed a good response to water vapor in the range of 12–86% RHs with a response time of 10–50 s and a recovery time of 70–100 s^26^. Zhang et al. reported that TiO_2_ nanotube arrays were calcined from 300 to 600 °C which showed the best sensitivity to humidity at 600 °C and had the response and recovery time of 100 s and 190 s, respectively^28^.

Since the power supply of humidity sensors through the battery reduces the capabilities of the sensor and increases environmental damage, one of the important applications of TENGs is the fabrication of humidity sensors. TENG charge density is highly affected by the surface variation of specified chemical molecules or environmental factor. Therefore, water vapor would considerably influence on the TENG output and we can see changes of ambient humidity through output current and voltage. This type of moisture sensors because of high flexibility can easily be connected to human organs and offer novel usage such as the expansion of electronic skin, health care and smart tracking^29,30^.

Here in this research, we have employed self-organized nanotubular TiO_2_ arrays as the flexible and also super-hydrophilic layer. The super-hydrophilicity of TiO_2_ nanotubes enhance the sensitivity of TENG-based humidity sensor since the moisture layer can influence the triboelectric charges on layers significantly; and also the flexibility of nanotubular layer makes this device easily practical. Nanotubular-based TENG reached a maximum external power of 1.25 ± 0.67 mW with the maximum Isc and Voc of 40.87 ± 1.74 µA and 307.77 ± 8.68 V at 4 Hz frequency, respectively. Furthermore, the output current is boosted by applying transformers up to 740.69 ± 24.08 µA. TiO_2_ nanotubular-based TENG employed as an active self-powered sensor that showed a good response to moisture with more than 300% change in rensponsivity. Increasing the relative humidity (RH) of the environment, gradually reduced the generated voltage from 162.24 ± 35.99 V and 20.4 ± 4.93 μA at RH = 20% to 37.92 ± 1.54 V at RH = 79% and 40.87 88 6.88 ± 1.7 μA at RH = 84%, respectively. This flexible battery-free TENG has potential application in diverse field of applications.

## Results and discussion

**Figure1:**
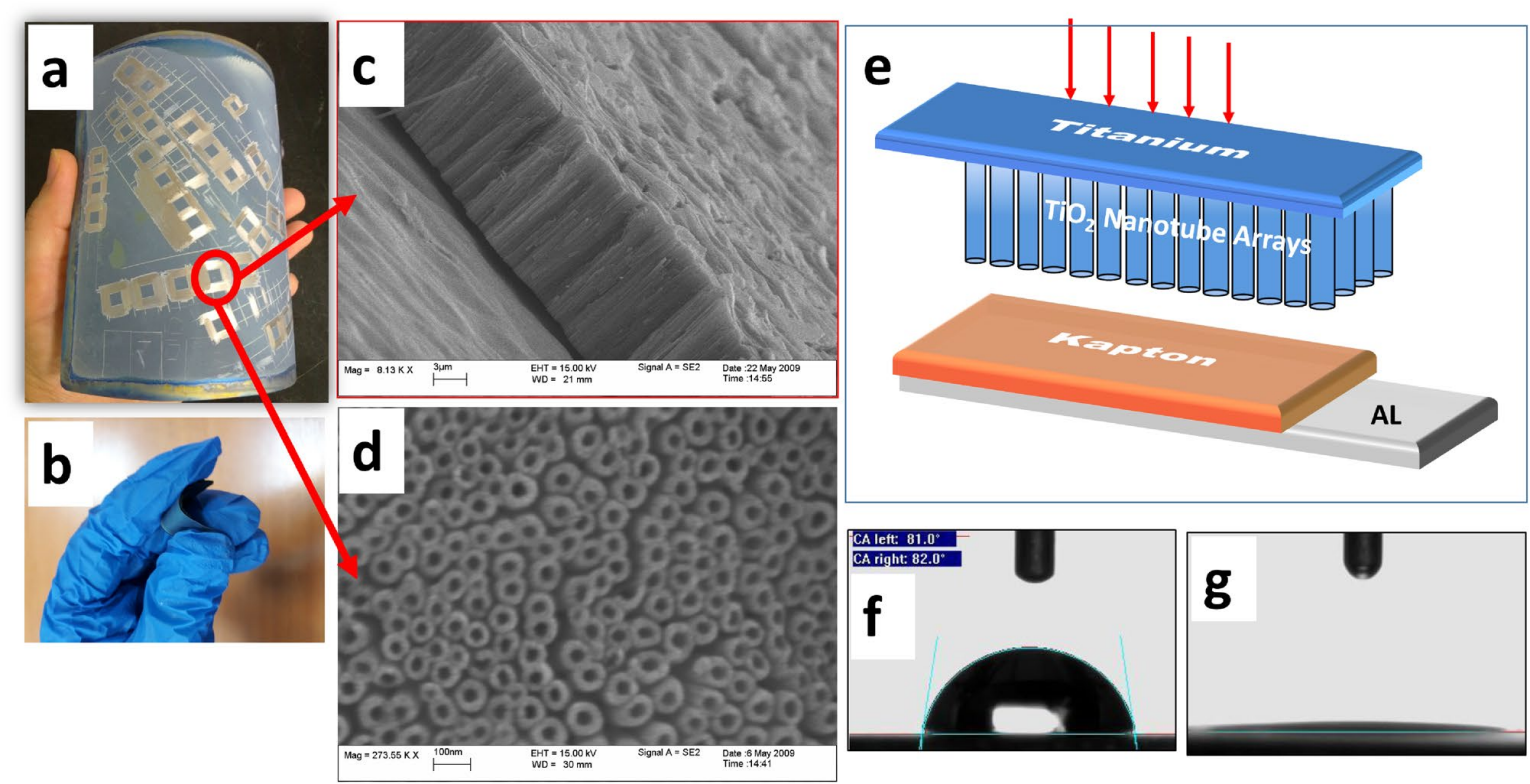
(**a**) 10 cm × 10 cm flexible TiO_2_ nanotube arrays of Ti foil fabricated through anodization method, (**b**) the flexibility of TiO_2_ layer, (**c**) cross section, (**d**) top view scanning electron microscopy image of anodized foil, (**e**) schematic of TENG consisted of TiO_2_ nanotube arrays on Ti and Kapton on Aluminium foil and contact angle measurement of 2 mL water drop on (**f**) Ti foil and (**g**) anodized Ti foil.

The flexible nanotubular TiO_2_ foil and the related SEM images are shown in Fig. 1a–c. The fabrication process resulted in production of flexible nanutubar film, 100 nm in diameter and 20 μm in length (Fig. 1c). TENG were fabricated from two layers and operated in vertical contact-separation mode. The static part was fabricated by attaching 2 Mil Kapton@Tape on the Aluminium tape, the contact provided directly through Aluminium tape (Fig. 1d). The moving parts are is Titanium nanotube array electrode. Electrodes had rectangular shape with the size of 3 cm × 3 cm. As it is obvious anodization of titanium foil resulted in superhydrophilic layers (contact angle ~ zero) which make it a suitable candidate for humidity sensor (Fig. 1e,f).

**Figure 2:**
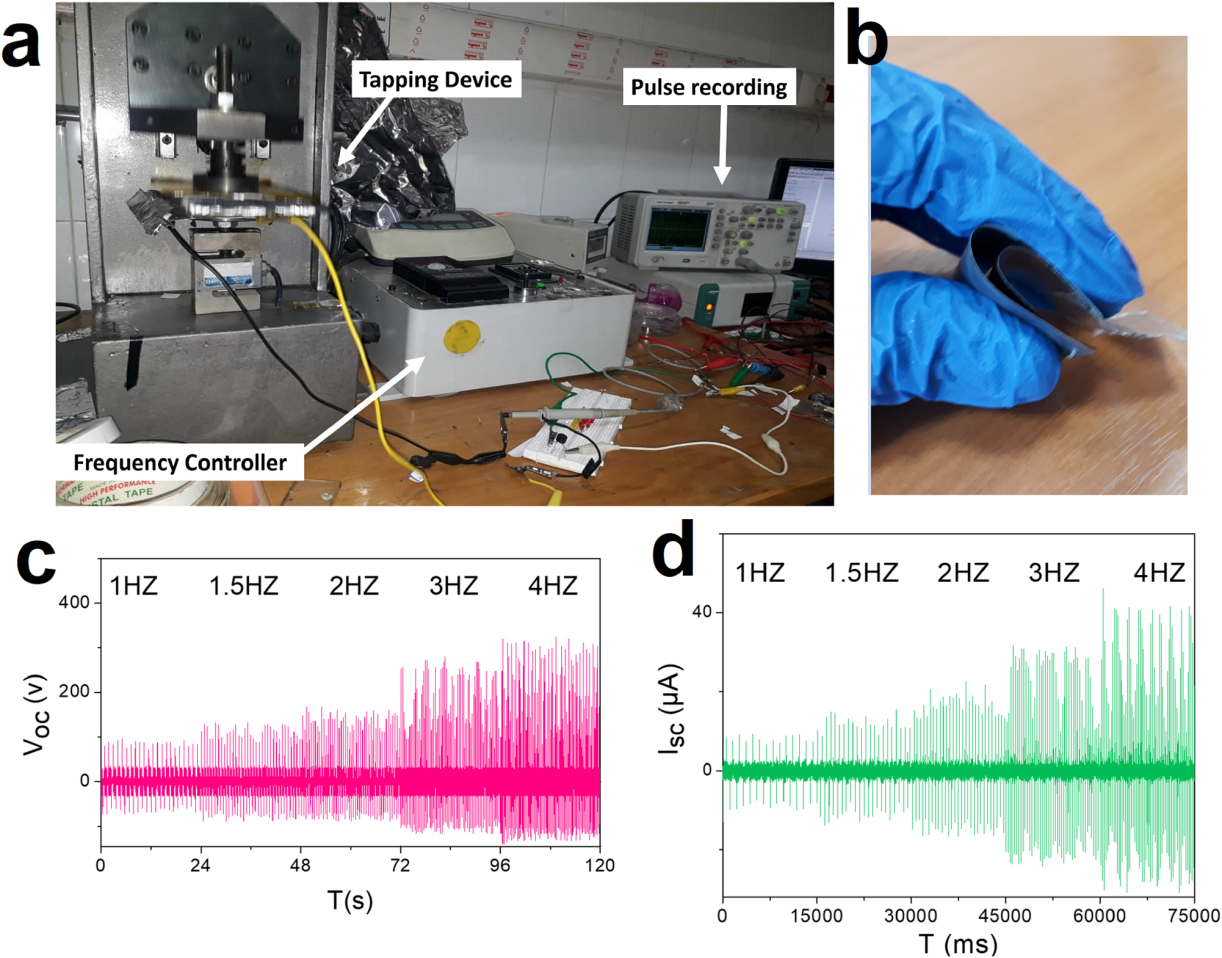
(**a**) The experimental setup for recording current and voltage upon tapping at various frequencies, (**b**) testing the flexibility of TENG device, (**c**) Open-circuit voltage, (**d**) Short circuit current, of the fabricated TiO_2_ TENG under frequencies from 1 to 4 Hz.

Figure 2a,b illustrate the home-made experimental setup for recording current and voltage upon tapping at various frequencies and also the flexibility of TENG device, respectively. Figure 2c,d demonstrate the open-circuit voltage and short-circuit current produced by TiO_2_-based TENG at various frequencies in the range of 1–4 Hz, respectively. According to the plotted diagram, the measured voltage and current at 1 Hz have the value of 87.75 ± 3.59 V and 7.15 ± 1.20 μA, which reaches 307.77 ± 8.68 V and 40.87 ± 1.74 μA at 4 Hz, respectively.

**Figure3:**
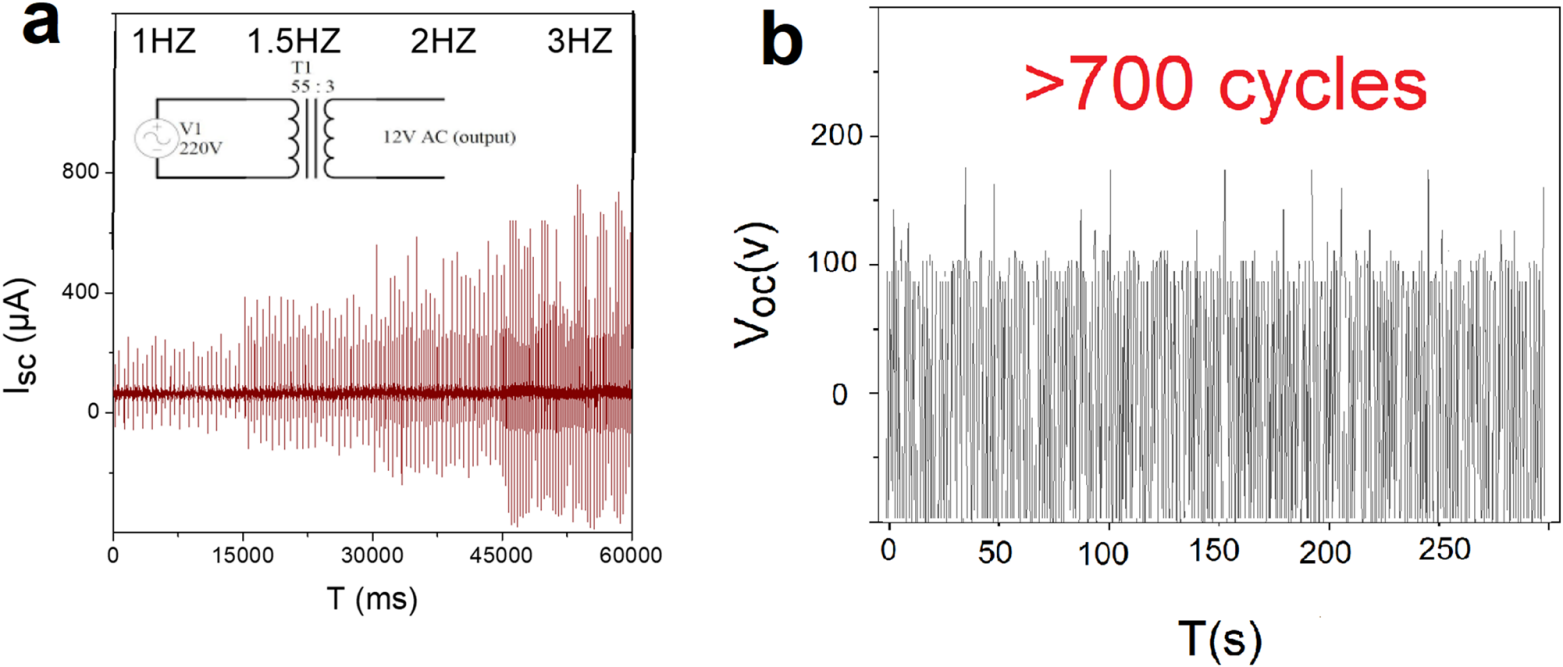
(**a**) Amplified output current at 1–3 Hz by utilizing 12 V transformer, (**b**) stability of the output voltage at 2 Hz.

By applying the 12 V-transformer, the short-circuit current at 3 Hz is also amplified to 740.69 ± 24.08 µA. (Fig. 3a). The stability of the generated voltage has been confirmed in Fig. 3b for over 700 cycles of tapping.

**Figure 4:**
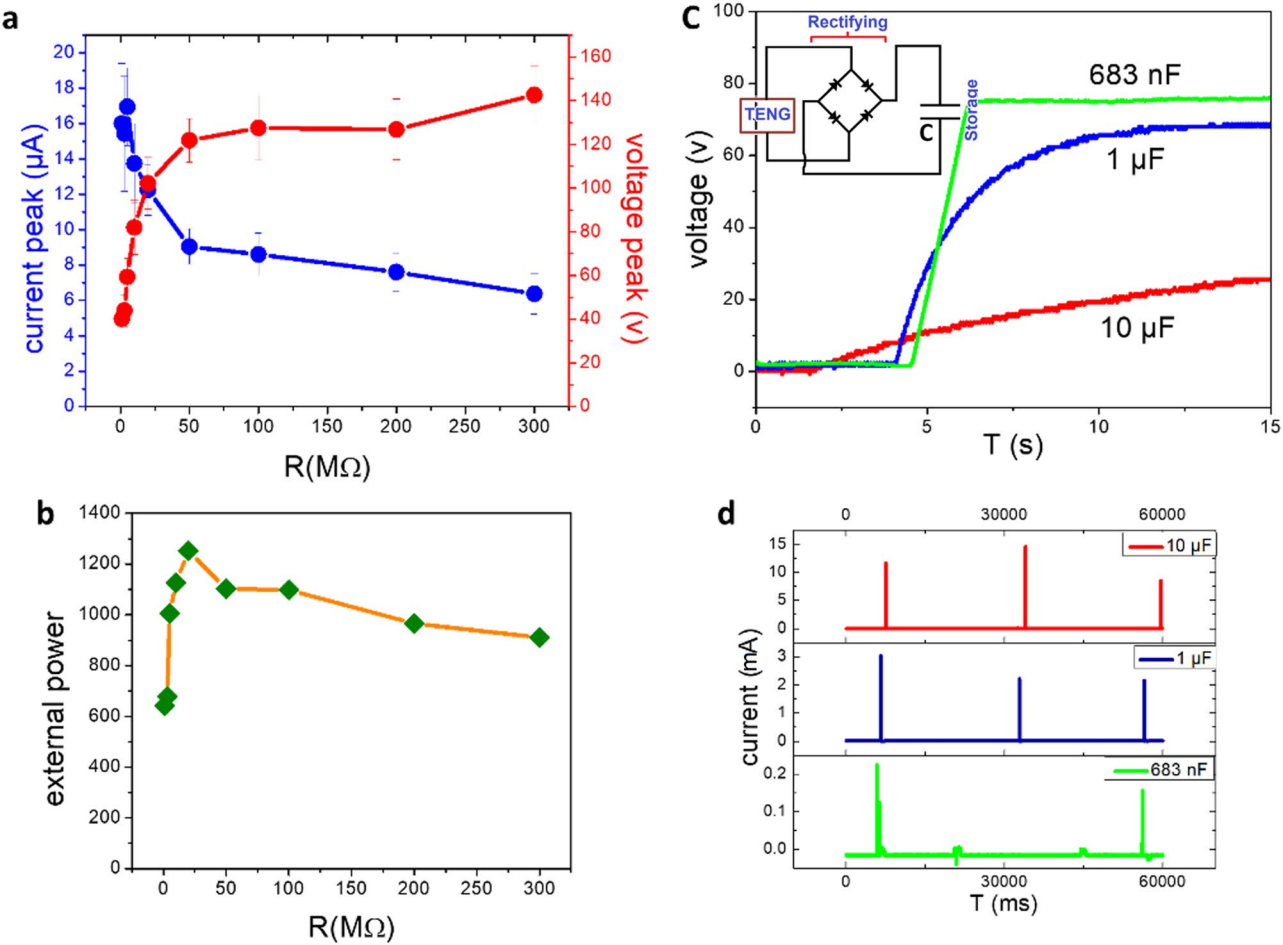
(**a**) Voltage and current peak of TiO_2_ TENG and (**b**) external power under different load resistances, (**c**) charge curve voltage and (**d**) current due to discharge of capacitors with different capacity by assistance of the TiO_2_ TENG.

Figure 4a demonstrates the average of voltage and current peak at various loading resistors. According to the equation Pmax = (VI) max, Voltage and current values are multiplied in each resistor to calculate the maximum power. The maximum power was obtained 1.25 ± 0.67 mW at 20 MΩ (Fig. 4b). As it is illustrated in this figure, with increasing loading resistor, the average of current decreases to near zero while the voltage peak increases to the open-circuit value.

TiO_2_-based TENG is also capable of charging a 683 nF and 1 μF capacitors in less than 6 and 12 s to the maximum voltage of 75.43 ± 4.17 V and 69.34 ± 1.05 V, respectively (Fig. 4c). In addition, the measured discharge current of capacitors with different capacities is shown in Fig. 4d indicates that the discharge current reaches ~ 200 μA during 2 s, 2 mA during 5 s and 13 mA during 15 s in the case of utilizing 683 nf, 1 μF and 10 µF capacitors respectively.

**Figure 5:**
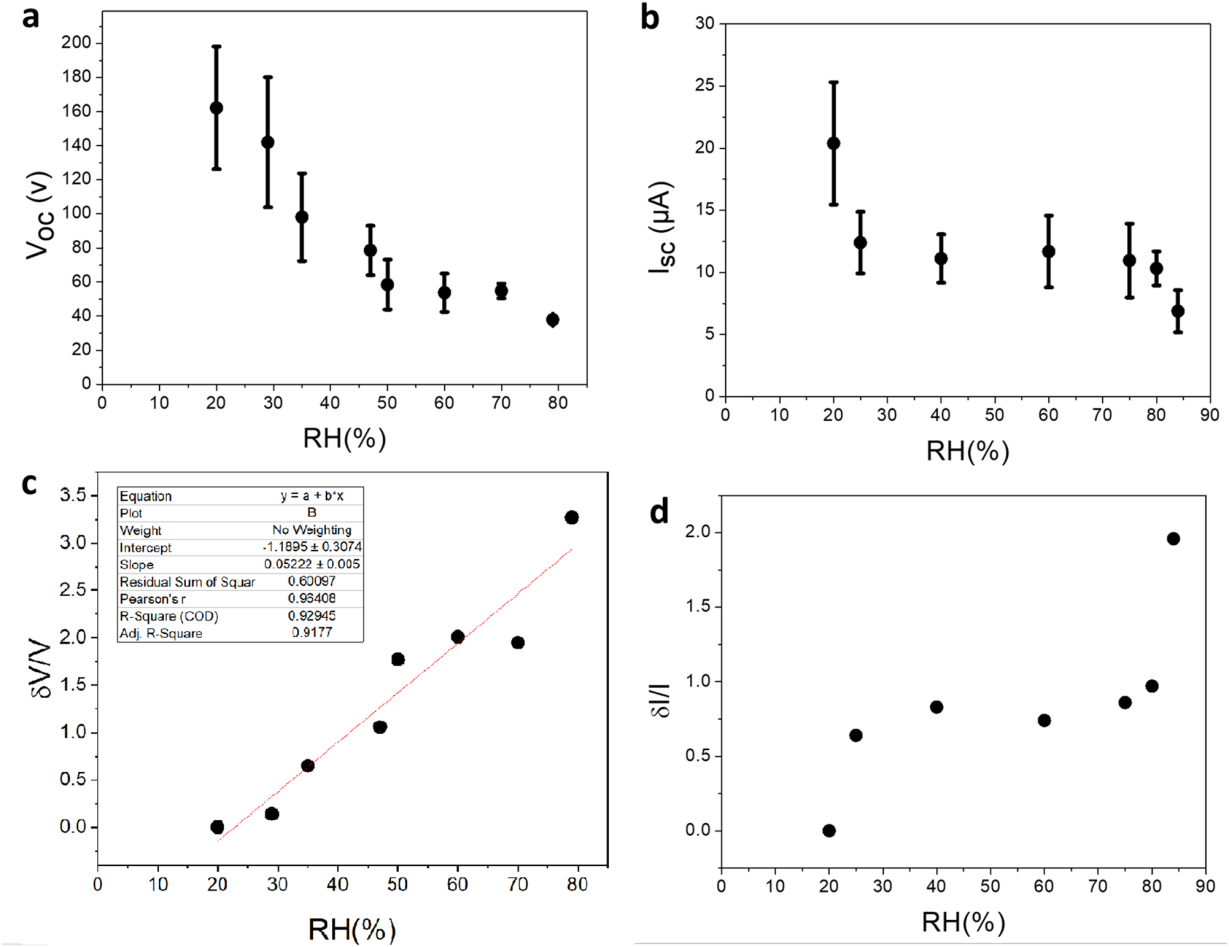
(**a**) Variation of the open-circuit voltage, (**b**) short-circuit current and (**c**,**d**) the corresponding response diagrams in different humidity RH.

The effect of ambient humidity on the voltage and current produced by TiO_2_ TENG have been examined (Fig. 5). Increasing the relative humidity percentage (% RH) increases the amount of water molecules adsorbed on the TiO_2_ surface and decreases the surface-induced charge through it, thus decreasing the output voltage and current as shown in Fig. 5a,b.

**Figure 6:**
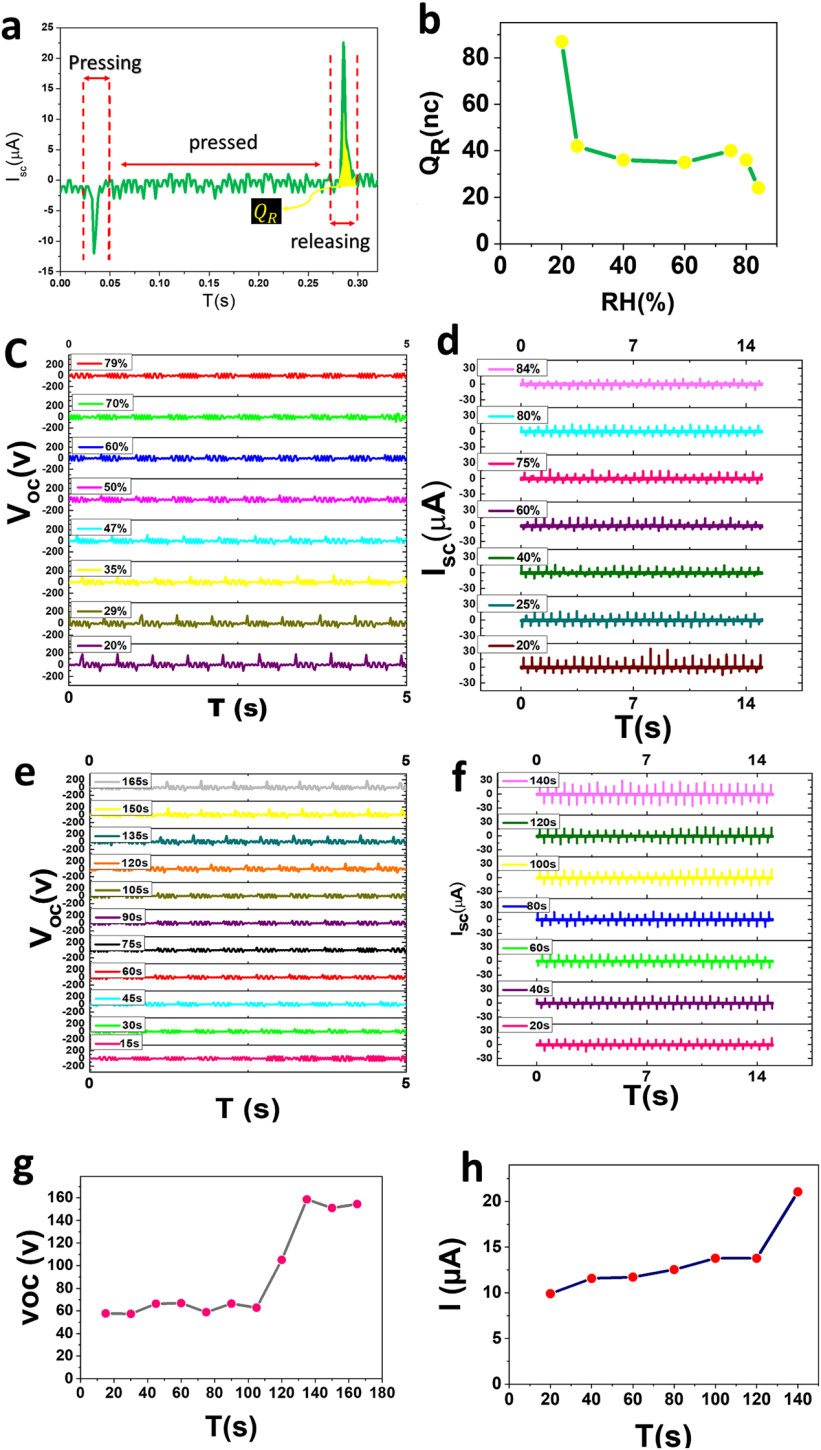
(**a**) The pressing and releasing peaks in a single tapping, (**b**) the amount of transferred charge versus RH during releasing, (**c**) variation of open-circuit voltage and (**d**) short-circuit current by increasing RH, (**e**) variation of open-circuit voltage and (**f**) short-circuit current and (**g**,**h**) the corresponding recovery time diagrams by disconnecting RH.

The performance of a sensor is determined by parameters such as response, response time, recovery time and repeatability. In order to accurately evaluate the data obtained, the voltage and current response diagrams of different relative humidity percentages (% RH) are plotted in Fig. 5c,d, respectively. Here, the response value for voltage and current is defined as (V0 − V)/V, while V0 and V corresponds to the voltage value at RH = 20 and the desired RH, respectively. The slope of response graphs generally refers to the sensitivity of the sensor. The slope of both response graphs increased with increasing RH. Steep of diagrams is significant at low and high RHs and is constant at middle of RHs. The response value of generated voltage reached to 320%, while the maximum value for current response is about 200% at RH = 85%. Based on the explored slope of response graph the sensitivity of sensor is about 0.05 ± 0.005/RH%. Resolution, which was called limit of detection in our previous response and might cause some vagueness, is reported here as the smallest change which is detectable by the sensor. Since the resolution of a sensor with a digital output is generally the resolution of that digital output, the value of resolution can be estimated by the resolution of the oscilloscope, which reports the voltage variation with the minimum value of 1 V. This amount of voltage is not proportional to a unique amount of RH in different regions of sensing diagram, changing from the value of 0.2% up to 1% RH. Therefore, the value of resolution can be reported as the worst one which is 1% RH for the whole range of detection. As it is illustrated in the diagrams of Fig. 6 all voltage pulse has been recorded in 5 s upon 10 tapping and the average values has been reported in curves of Fig. 5. As a result we can conclude the minimum of 5 s as the response time. Based on the diagrams of Fig. 6g,h, the recovery time is around 3 min.

Since water is a polar molecule, when the TiO_2_ is exposed to the humid environment, oxygen molecules that have negative charge are electrostatically adsorbed to TiO_2_ cations. To comprehend the relationship between the output voltage and current with the RH, the following adsorption process of water molecules on TiO_2_ surface.

At low RH, water vapor is physisorbed rapidly on the film surface. Hence, water molecules prevent the electrical induction between the negative charge on the kapton and the positive charge on the TiO_2_ film through reducing the contact surface, eventually causing a depletion region. Therefore, a rapid decline occurs in TiO_2_ TENG output current at low RHs. With increasing RH up to 70%, adsorption continues on the surface and on the pores walls.

By increasing the RH to over 70%, the porous area would be completely filled with water vapor molecules and a continuous layer of water vapor would be formed on the surface of TiO_2_. Thus, there would be a thorough depletion region, which occurs barricading the electrical induction between the two surfaces of the kapton and TiO_2_. Resulting would be shown a rapid decrease in output current at RHs of above 70%^31^.

Figure 6a illustrates the whole process of pressing-releasing for a single tapping. The surfaces under pressing and releasing peaks refer to the negative and positive charge transferred between the two electrodes. For calculating the amount of induced surface charge according to the equation of Q = ∫I·dt, it is obtained from the surface under the integral releasing peak. Figure 6b demonstrates the induced surface charge changes through increasing humidity. Increasing moisture reduces the surface transferred charge. As it is obvious by introducing humidity (up to 25%) to the nanotubular structure the transferred charge reduces noticeably, from 85 nc in RH 20–35 nc in RH 65%, that can be as the condensation of water molecule on the top of tubes. As the humidity increases in the range of 25–75% the humidity condenses in nanotubes, however the increase of humidity more than 75% results in full condensation on top of nanotubes and dramatic decrease in transferred charge has been detected. The output voltage and current changes of TENG TiO_2_ through increasing humidity has been shown in Fig. 6c,d, respectively. Voltage and current return to normal when the moisture released (Fig. 6e–h). These figures shows the reproducibility of sensor. Based on our result nanotubular TiO_2_ electrodes can be considered as the suitable electrode of TENG and also because of its super-hydrophilicity features it can be applied as the active electrode of self-powered humidity sensor.

In summary, a durable and flexible TiO_2_ TENG with maximum output power density 1.25 ± 0.67 mW at 4 Hz frequency has been fabricated through a straightforward method. Due to its hydrophilic, TiO_2_ absorbs the water molecules with increasing moisture, and the charge transfer decreases between the two triboelectric materials as the surface charge transfer depends on the adsorbent molecules. The results demonstrated that with increasing relative humidity (RH) the output voltage and current shows the responsivity more than 300%. This method of measuring humidity has a simple and cost-effective fabrication that has various applications in many fields such as industry and medicine.

## Methods

TiO_2_ nanotube arrays have been fabricated by anodization of (10 cm × 10 cm), 250 µm Titanium foil (purity 99.5%; Alfa Aesar) in ethylene glycol containing 0.2 vol% H_2_O and 0.3 M NH_4_F at 60 V bias voltage in two-electrode electrochemical setup for 100 min at room temperature to get nanotubes with the length of 20 µm. Nanotubes were annealed at 450 °C for 6 h in pure oxygen with heating and cooling rates of 1 °C min^−1^ to get pure anatase phase. The precise method for fabrication and characterization of self-organized TiO_2_ nanotubes can be found in our previous publications^32,33^. The Wettability has been investigated utilizing Dataphysics-OCA setup by recording the contact angle of 2 µL drop on nanotube surface. TENG were fabricated from two layers and operated in vertical contact-separation mode. The static part was fabricated by attaching 2 Mil Kapton@Tape on the Aluminium tape, the contact provided directly through Aluminium tape. The moving parts are is Titanium nanotube array electrode. Electrodes had rectangular shape with the size of 3 cm × 3 cm. The current characterizations were performed using autolab potentiostat (Metrohm Autolab PGSTAT 302N) and the acquired voltages were recorded using 2 GHz Agilent digital oscilloscope. All experiments has been done by home-made atuomatic tapping instrument with the control of force and frequency of tapping.

## Data Availability

Derived data supporting the findings of this study are available from the corresponding author on request.
